# Effect of Fatty Acids on Human Bone Marrow Mesenchymal Stem Cell Energy Metabolism and Survival

**DOI:** 10.1371/journal.pone.0120257

**Published:** 2015-03-13

**Authors:** Natasha Fillmore, Alda Huqi, Jagdip S. Jaswal, Jun Mori, Roxane Paulin, Alois Haromy, Arzu Onay-Besikci, Lavinia Ionescu, Bernard Thébaud, Evangelos Michelakis, Gary D. Lopaschuk

**Affiliations:** Cardiovascular Research Centre, Mazankowski Alberta Heart Institute, University of Alberta, Edmonton, Alberta, Canada; Georgia Regents University, UNITED STATES

## Abstract

Successful stem cell therapy requires the optimal proliferation, engraftment, and differentiation of stem cells into the desired cell lineage of tissues. However, stem cell therapy clinical trials to date have had limited success, suggesting that a better understanding of stem cell biology is needed. This includes a better understanding of stem cell energy metabolism because of the importance of energy metabolism in stem cell proliferation and differentiation. We report here the first direct evidence that human bone marrow mesenchymal stem cell (BMMSC) energy metabolism is highly glycolytic with low rates of mitochondrial oxidative metabolism. The contribution of glycolysis to ATP production is greater than 97% in undifferentiated BMMSCs, while glucose and fatty acid oxidation combined only contribute 3% of ATP production. We also assessed the effect of physiological levels of fatty acids on human BMMSC survival and energy metabolism. We found that the saturated fatty acid palmitate induces BMMSC apoptosis and decreases proliferation, an effect prevented by the unsaturated fatty acid oleate. Interestingly, chronic exposure of human BMMSCs to physiological levels of palmitate (for 24 hr) reduces palmitate oxidation rates. This decrease in palmitate oxidation is prevented by chronic exposure of the BMMSCs to oleate. These results suggest that reducing saturated fatty acid oxidation can decrease human BMMSC proliferation and cause cell death. These results also suggest that saturated fatty acids may be involved in the long-term impairment of BMMSC survival *in vivo*.

## Introduction

The potential for stem cell therapy to regenerate injured tissue has recently generated considerable interest. Two major problems facing stem cell heart therapy include low stem cell survival *in vivo* and negligible stem cell-to-target cell differentiation *in vivo* [1–6]. The development of strategies to solve these problems should be facilitated by a better understanding of stem cell biology. One aspect of this biology that we believe will be particularly important to better understand is the regulation of energy metabolism because of its potential importance in differentiation and cell proliferation, important characteristics of stem cells [7–12].

The concept that energy metabolism is involved in mediating cell proliferation was first introduced by Otto Warburg. His finding, referred to as the Warburg effect, was that highly proliferative cancer cells have high rates of glycolysis even under aerobic conditions [13,14]. The survival and proliferation of these highly glycolytic cells correlate with high glycolysis rates [15]. Increasing the coupling of glycolysis to glucose oxidation by treating cancer cells with dichloroacetate, a drug that increases pyruvate dehydrogenase (PDH) activity by inhibiting pyruvate dehydrogenase kinase (PDK), not only increases glucose oxidation but also decreases glycolysis, decreases proliferation, and increases apoptosis [9]. Genetically decreasing PDK expression also increases overall oxidative metabolism and decreases the proliferation of cancer cells [9,16]. While not identical, embryonic stem cells (ESCs) and embryonal carcinoma cells have similar levels of metabolites, especially those involved in glycolysis [17]. Therefore, cancer cell metabolism may provide a clue to the metabolism of stem cells. While there is relatively little evidence, the data do indicate that high glycolysis and low oxidative metabolism is important in stem cell survival and proliferation [18–21].

Glycolysis is believed to be important in proliferation because it provides the cell with substrates needed to maintain high rates of macromolecular synthesis. For example, lipogenesis requires NADPH, which is produced by the pentose phosphate cycle that temporarily shunts substrates away from glycolysis. NADPH production and its use in lipogenesis appears to be essential for cancer cell proliferation [22,23]. In addition, a key transcription factor regulating glycolysis, hypoxia inducible factor 1α (HIF1α), enhances macromolecular synthesis by increasing the protein expression of isocitrate dehydrogenase (IDH) 2 [24]. IDH2 helps convert α ketoglutarate back to citrate which can be transported out of the mitochondria and used in lipogenesis.

The concept that high glycolysis and low oxidative metabolism is necessary for proliferation and survival of proliferating cells is not completely straightforward. For example, stimulation of fatty acid oxidation protects glioblastoma cells, which are normally dependent on Akt for anaerobic glycolysis and survival, from death induced by glucose deprivation [25]. It has also been shown that expression of carnitine palmitoyltransferase 1c, a protein involved in mitochondrial fatty acid transport, or uncoupling protein 2 (UCP2) protects cancer cells from hypoxia and glycolysis inhibition by providing an alternative pathway for energy production [11,26]. This capacity for fatty acid oxidation to maintain cancer cell proliferation and survival is not true for all cancer cells and may be unique to cancer cells. These findings do suggest that oxidative metabolism, and specifically fatty acid oxidation, does not always hinder proliferative cell survival.

Despite the potential importance of glycolysis and fatty acid oxidation on stem cell viability and proliferation, very little is known about the control of energy metabolism in stem cells. Indeed, very little is known about the viability of stem cells exposed to the concentrations of fatty acids normally seen *in vivo*. We therefore characterized bone marrow mesenchymal stem cell (BMMSC) energy metabolism and investigated the effect of fatty acids on BMMSC metabolism and survival. We report here the first direct energy metabolic rate profile of BMMSCs, confirming that BMMSCs are highly glycolytic. We also examined what effect physiological levels of fatty acids present in the circulation have on BMMSC glucose and fatty acid metabolism and survival. We demonstrate *in vitro* that fatty acids induce BMMSC death which suggests that fatty acids may be involved in the low survival observed in stem cells *in vivo* and that an understanding of the effect of fatty acids on BMMSC metabolism is important in developing strategies to successfully augment stem cell survival.

## Materials and Methods

### Cell culture

Human BMMSCs were used in this study. Standard cell culture procedures were used. Human BMMSCs were treated with media containing low glucose α-MEM, 16.5% fetal bovine serum (FBS), 1% glutamine, and 1% streptomycin/penicillin. Cells were cultured at 37°C and 5% CO_2_. During experiments assessing the chronic effect of fatty acids on BMMSCs this media was also supplemented with 4% fatty acid free bovine serum albumin (BSA) (Equitech-Bio Inc BAH66) or 4% BSA bound to the indicated type and concentration of fatty acid (Palmitate, Sigma P9767; Oleate, Fluka Analytical 60420; Stearate, Sigma S3381). For experiments assessing the acute effect of fatty acids, cells were exposed to the normal media described above until beginning measurement of metabolism. More information on this assay and the media used that was supplemented with BSA and fatty acids is provided below in the metabolic rates and fatty acid uptake section. Human BMMSCs from a single donor were purchased from the Texas A&M Health Science Center already characterized. Some of the measurements involved in this characterization included confirmation of ability to undergo adipogenesis and osteogenesis, expression of CD105, CD73, and CD90, and absence of CD45, CD34, and CD14 expression. Human BMMSCs were passaged at 70% confluency with 60 cells seeded per cm^2^.

### MTT assay

A standard MTT assay protocol was used to assess cell viability. Briefly, 0.5 mg/ml MTT (Invitrogen M6494) was added to aspirated wells for 2 hr at 37°C. Wells were then aspirated, rinsed with PBS and the product, formazan, was dissolved in DMSO. If necessary, 200 μl were transferred to a 96 well plate. Absorbance was measured at 550 nm.

### Caspase activity assay

Caspase activity was measured using a DEVD-AMC (Sigma A1086) kit. The standard procedure provided by Sigma was used.

### Immunofluorescence

Standard immunofluorescence methods were used. Images were taken with the confocal microscope Zeiss LSM 510 NLO. Terminal deoxynucleotidyl transferase dUTP nick end labeling (TUNEL) and Ki67 staining in fixed cells was performed as described previously [27]. Mitochondrial membrane potential was measured in live cells with TMRM staining as described previously [9]. The average intensity of mitochondrial membrane potential was assessed using Zeiss LSM 510 software.

### Western blots

Western blotting was performed using standard procedures. Briefly, samples were loaded into wells in Tris-HCl gels and run at 60V for 10 min initially and then switched to 120V. Protein in the gel was then transferred onto nitrocellulose at 90 V for 2 hr. Membranes were then blocked for 1 hr in 5% non-fat dry milk (NFDM) in TBST, probed overnight at 4°C with primary antibody, rinsed 4 x 5 min in TBST, probed with appropriate secondary antibody for 1 hr at room temperature, and then rinsed in TBST 4 x 5 min. Primary antibodies included HIF1α (Novus Biologics, NB100–105), LDH-A (Santa Cruz sc-27230), IDH (Abcam, ab36329), phosphoglycerate mutase 1 (PGAM1) (Cell Signaling 7534S), cyclin D1 (Cell Signaling 2922), phospho Rb S780 (Cell Signaling 9307S), phospho acetyl CoA carboxylase (ACC) (Millipore 07–303), and ACC (Jackson 016–050–084). The secondary antibodies used with appropriate primary antibody included anti rabbit (Santa Cruz, sc-2054), anti mouse (Santa Cruz, sc-2055), or anti goat (Santa Cruz, sc-2056). Chemiluminescent detection was then performed using enhanced chemiluminescence (ECL) and was detected using autoradiography film. Western blots were analyzed using Image J. Ponceau red staining was used to correct for any variation in protein loading between samples. Values presented in graphs were normalized against the BSA group.

### Metabolic rates and fatty acid uptake

Glycolysis, glucose oxidation, palmitate oxidation, and oleate oxidation were measured in cells grown in T25 flasks. At the beginning of each of these assays, cell culture media was switched out for Krebs Henseleit buffer (118 mM NaCl, 4.7 mM KCl, 1.2 mM KH_2_PO_4_, 1.2 mM MgSO_4_7H_2_O, 2.5 mM CaCl_2_2H_2_O, 25 mM NaHCO_3_) supplemented with at least 5 mM glucose, 0.55 mM fatty acid free BSA (Equitech-Bio Inc BAH66) and the appropriate radioactive labeled fatty acid or glucose (as indicated below). The presence of 0.4 mM palmitate (Sigma P0500) and/or 0.4 mM oleate (Fluka Analytical 60420) in the Krebs Henseleit buffer is indicated in the figure legends. Because fatty acids are normally bound to albumin in the blood, and the actual concentration of free fatty acid levels that the cell is exposed to depends on the fatty acid to albumin ratio [28], all metabolic measurements were performed with 0.55 mM albumin, which is the concentration of albumin normally seen in the blood. Fatty acids were bound to albumin. Glycolysis was measured using cells incubated with [5–^3^H]glucose and the ^3^H_2_O released at the enolase step of glycolysis was measured. Glucose oxidation, palmitate oxidation, and oleate oxidation was measured using [U-^14^C]glucose, [1–^14^C]palmitate, or [1–^14^C]oleate, respectively. ^14^CO_2_ formed was captured over a period of 3 hr. For ^3^H_2_O detection, following addition of [5–^3^H]glucose flasks were kept at 37°C for 2 hr. Duplicate 200 μl samples of the media were then transferred from each flask into 1.5 ml capless centrifuge tubes placed inside a scintillation vial with 500 μl of ddH_2_O at the bottom of the vial. ^3^H_2_O standard and the unmetabolized buffer were also added in parallel. Capped scintillation vials were left at 50°C for 24 hr and then transferred to 4°C overnight. Care was taken to keep any H_2_O on the sides of each tube inside the scintillation vials. In duplicate, 200 μl of the ^3^H_2_O standard and unmetabolized buffer were also placed into empty scintillation vials in order to calculate transfer efficiency and specific activity. For ^14^CO_2_ detection the flask was attached to a CO_2_ capture device and placed in a dry incubator at 37°C for 3 hr. ^14^CO_2_ in the media was released by adding 1 ml of 9 M sulphuric acid to the flask with a needle through a rubber stopper in order to not compromise the closed system. CO_2_ was captured using a hyamine hydroxide soaked filter paper at the top of the CO_2_ capture device. The CO_2_ capture device has been previously described [29]. If fatty acid uptake was measured instead of oxidation, cells were washed three times with PBS. Following homogenization, the supernatant was counted to determine the amount of palmitate uptake. All hyamine soaked filter papers from the flasks were placed in scintillation vials. Scintillation fluid (Fisher, SX23–5) was added to all scintillation vials and counted in a scintillation counter (Perkin Elmer, 2800TR). Rates of ATP production from energy metabolism were calculated based on 2 ATP produced/ molecule of glucose passing through glycolysis, 30 ATP for each molecule of glucose oxidized, and 105 ATP for each palmitate molecule oxidized.

### Statistical analysis

Values are presented as mean ± SEM. One-way ANOVA followed by Bonferroni's Multiple Comparison Post Hoc Test or t-test was performed using Prism software to determine statistical significance. Results shown in figures are from combined experiments. Number of experimental replicates is indicated in figure legends. Differences are considered statistically significant if p˂0.05. Values are presented as mean ± SEM.

## Results

### Profile of human BMMSC energy metabolism

We report here the first direct measurements of energy metabolic rates in BMMSCs (Table 1). These studies were performed under experimental conditions in which cells were incubated with concentrations of glucose (5 mM), fatty acids (0.4 mM palmitate), and albumin (0.55 mM). Glucose and albumin levels mimic the concentrations of these substrates normally present in the blood. The normal circulating level of fatty acids under non-fasting conditions is 0.4 mM while the concentration of the fatty acid palmitate is about 0.2 mM. Palmitate was used as the representative fatty acid in this assay. In human BMMSCs glycolysis rates (494±33 nmol/mg protein/hr) are extremely high compared to glucose oxidation (0.79±0.22 nmol/mg protein/hr) and palmitate oxidation rates (0.02±0.005 nmol/mg protein/hr). From these rates ATP production from each of these pathways was calculated: glycolysis (988.2±66.7 nmol ATP/mg protein/hr), glucose oxidation (23.8±6.5 nmol ATP/mg protein/hr), and palmitate oxidation (2.07±0.51 nmol ATP/mg protein/hr) (Table 1). Based on these rates, 97.5% of ATP in the BMMSCs is derived from glycolysis, 2.2% of ATP from glucose oxidation, and 0.2% of ATP from palmitate oxidation. This confirms the previous assumptions that stem cells are primarily deriving their energy from glycolysis.

**Table 1:** Contribution of energy metabolism pathways to ATP production in human BMMSCs.

	Metabolism Rate nmol/mg protein/hr	ATP Production nmol/mg protein/hr	ATP Production %
Glycolysis	494±33	988.2 ± 66.67	97.5 ± 0.67
Glucose Oxidation	0.79±0.22	23.8 ± 6.50	2.2 ± 0.63
Palmitate Oxidation	0.02±0.005	2.07 ± 0.51	0.2 ± 0.05

During each assay Krebs Henseleit buffer was supplemented with 5 mM glucose and 0.4 mM palmitate bound to 0.55 mM albumin. In addition, the Krebs Henseleit buffer was supplemented with either [U-^14^C]glucose, [1–^14^C]palmitate, or [5–^3^H]glucose in order to measure glucose oxidation, palmitate oxidation, or glycolysis, respectively. Bone marrow mesenchymal stem cells (BMMSCs) were exposed to normal cell culture media immediately up to the point it was switched to this Krebs Henseleit buffer at the start of each assay. Calculations to determine ATP production and % ATP production were made from the metabolic rate results. n = 5–8 Values are shown as the mean ± SEM.

### Fatty acids affect BMMSC survival

**Fig 1:**
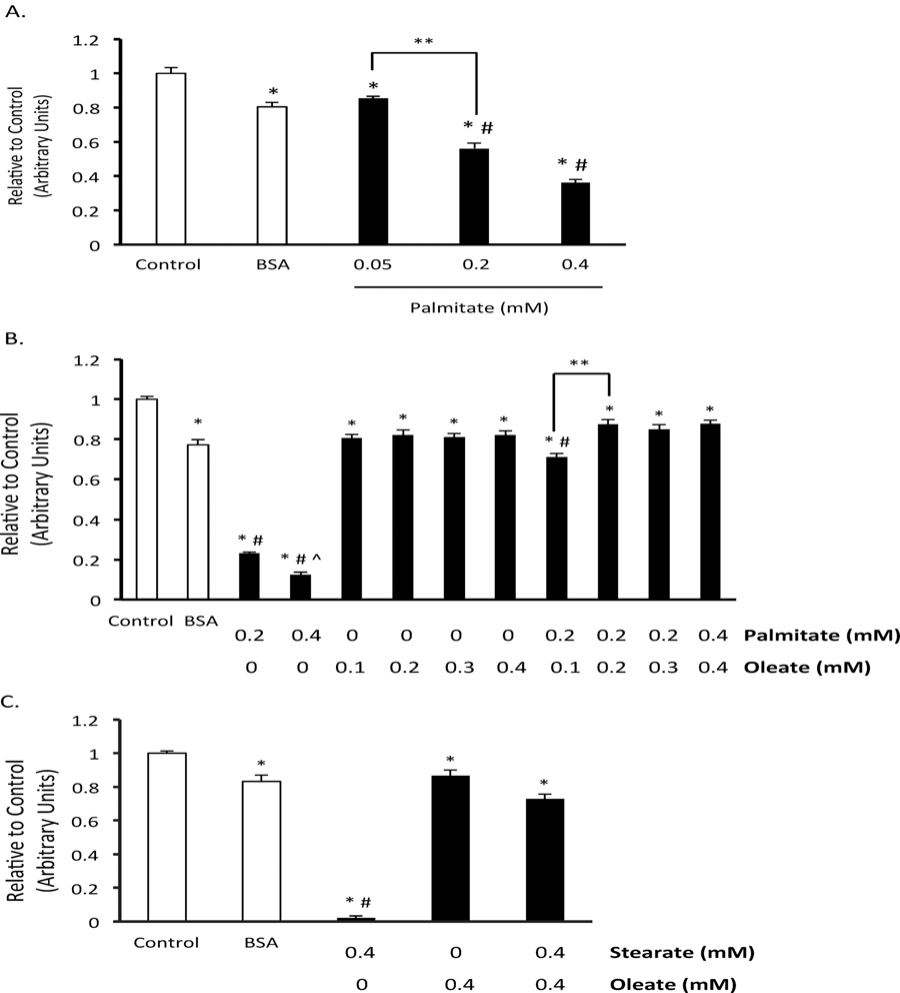
Oleate prevents palmitate-induced human BMMSC death. A) Effect of palmitate on human bone marrow mesenchymal stem cell (BMMSC) viability was measured by the MTT assay after 48 hr treatment with indicated treatments. B) Effect of the ratio of palmitate and oleate on palmitate-induced BMMSC death after 72 hr treatment was measured by MTT assay. C) Effect of 72 hr treatment with stearate and/or oleate on the amount of viable cells was measured by the MTT assay. The BSA group was treated with media supplemented with 0.55 mM albumin. All fatty acid treated groups were also treated with media supplemented with 0.55 mM albumin in addition to the type and amount of fatty acid indicated in the figures. The Control group was treated with media identical to the BSA group minus supplementation with albumin. n = 7–12 * Significantly different from Control group. # Significantly less than BSA group. ^ Significantly less than 0.2 mM Palmitate Group. ** Groups are significantly different. Values are shown as the mean ± SEM.

When stem cells are introduced into the target organ they become exposed to the blood. Some of the blood’s contents include glucose, fatty acids, and albumin. Surprisingly, a survey of cell culture media indicate that the level of fatty acids and albumin are much lower than what is present in the circulation [30]. We, therefore, were interested in what effect fatty acids might have on BMMSC viability. When we exposed BMMSCs to media supplemented with levels of palmitate (0.05–0.4 mM) and albumin (0.55 mM) normally present in the blood, we noticed that palmitate in a concentration and time dependent manner decreased BMMSC viability (Fig. 1). In contrast, exposure of the BMMSCs to only 0.55 mM albumin did not result in any major decrease in cell viability (Fig. 1). Importantly, not only pathological levels (0.4 mM), but also normal circulating levels of palmitate (0.2 mM) decreased BMMSC viability (Fig. 1). Further, another saturated fatty acid, stearate, also decreased BMMSC viability (Fig. 1c). Because both unsaturated and saturated fatty acids are present in the circulation, we also examined what effect the unsaturated fatty acid oleate, the most abundant fatty acid in the circulation, has on BMMSC viability. When we treated BMMSCs with physiologically relevant levels of the unsaturated fatty acid oleate (bound to 0.55 mM albumin) we did not observe a change in BMMSC viability (Fig. 1b). Further, if palmitate treated BMMSCs were also exposed to oleate, BMMSC viability was protected (Fig. 1). Oleate also protected against stearate-induced BMMSC death. These results indicate that saturated fatty acids could be a contributing factor in the loss in viability observed when stem cells are introduced into the body for therapeutic purposes.

### Palmitate induces apoptosis and decreases proliferation

**Fig 2:**
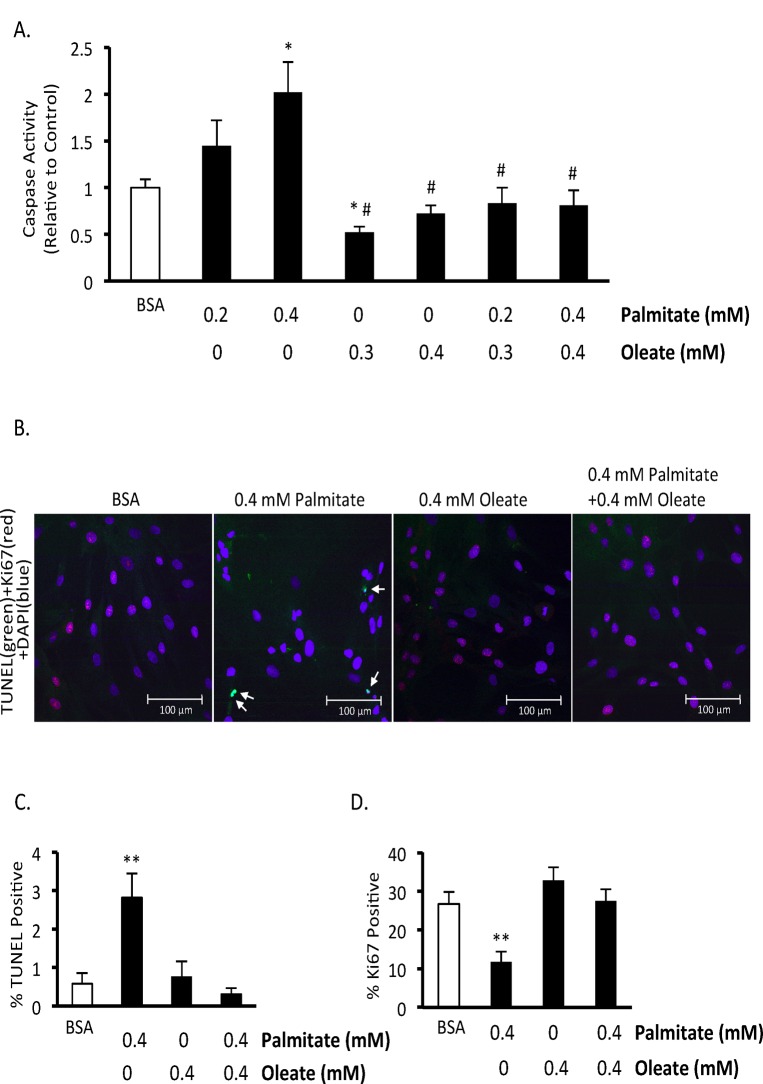
Oleate prevents palmitate-induced human BMMSC apoptosis and reduction in proliferation. A) Caspase activity after 48 hr treatment with indicated treatments. B) Images of terminal deoxynucleotidyl transferase dUTP nick end labeling (TUNEL) and Ki67 staining of bone marrow mesenchymal stem cells (BMMSCs) treated for 24 hr with indicated treatments. Image is 28X. C) % nuclei positive for TUNEL. D) % nuclei positive for Ki67. n = 5–7 The BSA group was treated with media supplemented with 0.55 mM albumin. All fatty acid treated groups were also treated with media supplemented with 0.55 mM albumin in addition to the type and amount of fatty acid indicated in the figures. * Significantly different from BSA group. # Significantly different from 0.2 mM Palmitate and 0.4 mM Palmitate groups. ** Significantly different from all groups. Values are shown as the mean ± SEM.

**Fig 3:**
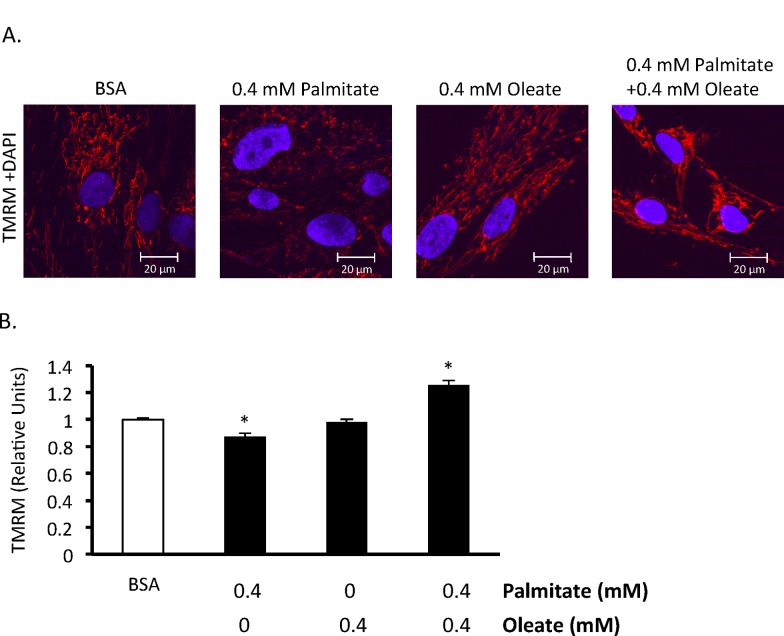
Effect of 24 hr exposure to palmitate and oleate on human BMMSC mitochondrial membrane potential. A) Images of tetramethylrhodamine methyl ester (TMRM) stained bone marrow mesenchymal stem cells (BMMSCs) treated for 24 hr with indicated treatments. B) Relative TMRM levels. BMMSCs were treated for 24 hr with 0.55 mM bovine serum albumin (BSA) alone or palmitate and/or oleate bound to 0.55 mM albumin. TMRM and Hoechst stain were added to the medium to measure mitochondrial membrane potential and stain nuclei, respectively, and images were taken. All fatty acid treated groups were also treated with media supplemented with 0.55 mM albumin in addition to the type and amount of fatty acid indicated in the figures. 4 separate observations. Values are shown as the mean ± SEM. * Significantly different from the BSA group.

To confirm that palmitate induces BMMSC death and to assess whether the type of cell death involves apoptosis, we measured mitochondrial membrane potential, performed TUNEL staining, and assessed caspase 3 activity. Chronic exposure of BMMSCs to palmitate (48 hr exposure) resulted in an increase in caspase 3 activity (Fig. 2A). Palmitate also increased TUNEL staining after 24 hr (Fig. 2B,C) and decreased mitochondrial membrane potential (Fig. 3A,B).

We also investigated the effect of palmitate on BMMSC proliferation. To do this, we assessed the nuclear expression of Ki67, a marker of proliferation. There is a lower percentage of Ki67 positive human BMMSC nuclei following palmitate exposure (Fig. 2D).

### Oleate inhibits palmitate-induced human BMMSC apoptosis and reduction in proliferation

To examine the relationship between saturated and unsaturated fatty acids on BMMSC viability we treated BMMSCs with varying ratios of palmitate and oleate up to a normal physiological range (0.2 mM palmitate and 0.3 mM oleate) during non-fasting conditions. When BMMSCs are exposed to equal or greater amounts of oleate BMMSC viability is preserved (Fig. 1B,C and Fig. 2A,C). At the lower ratio tested (0.1 mM oleate and 0.2 mM palmitate) oleate is only partially protective. In addition, oleate prevents the increase in caspase activity and TUNEL positive nuclei induced by palmitate treatment (Fig. 2A,B). Oleate also protects against the drop in mitochondrial membrane potential induced by palmitate (Fig. 3A,B). Finally, oleate prevents the drop in Ki67 positive human BMMSCs following 24 hr of palmitate treatment (Fig. 2D). We also looked to see if cyclin D1/Rb signaling, which can regulate proliferation and has been shown to be affected by hematopoietic stem cell exposure to palmitate [31], is involved in this drop in proliferation. However, phosphorylation of S780 Rb and total cyclin D1 protein expression were not significantly affected following 24 hr treatment with palmitate and/or oleate (data not shown).

### Acute effect of the fatty acids palmitate and oleate on BMMSC energy metabolism

**Fig 4:**
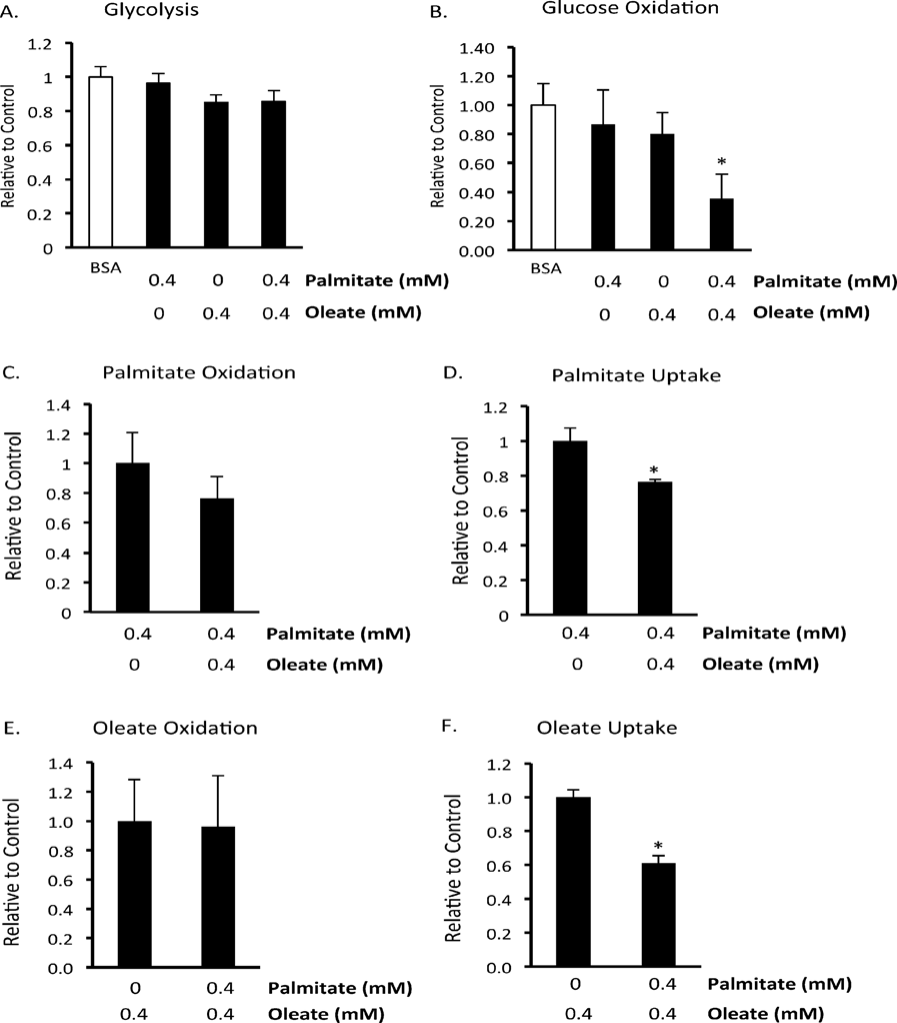
Effect of acute exposure to fatty acids on human BMMSC energy metabolism. A) Glycolysis, B) glucose oxidation, C) palmitate oxidation, D) palmitate uptake, E) oleate oxidation, and F) oleate uptake were measured in untreated human bone marrow mesenchymal stem cells (BMMSCs). n = 5–7 During each assay Krebs Henseleit buffer was supplemented with 5 mM glucose and, as indicated in each graph, either 0.55 mM albumin (BSA group) or 0.55 mM albumin bound to 0.4 mM palmitate and/or 0.4 mM oleate. In addition, the Krebs Henseleit buffer was supplemented with either [U-^14^C]glucose, [1–^14^C]palmitate, [1–^14^C]oleate, or [5–^3^H]glucose for the measurement of glucose oxidation, palmitate oxidation and uptake, oleate oxidation and uptake, or glycolysis, respectively. Since these experiments assessed the acute effect of palmitate and oleate, BMMSCs were maintained in cell culture media used to culture these immediately up to the start of each assay when the media was switched to Krebs Henseleit buffer supplemented with fatty acids. The levels and type of fatty acid BMMSCs were exposed to is indicated on the x-axis of the figures. * Significantly different from all groups. Values are shown as the mean ± SEM.

To determine whether alterations in energy metabolism could be involved in palmitate-induced cell death we assessed the acute effects of palmitate and oleate on BMMSC energy metabolism. Glycolysis, glucose oxidation and fatty acid oxidation were measured in BMMSCs treated with 5 mM glucose and either 0.55 mM BSA, 0.4 mM palmitate bound to 0.55 mM BSA, 0.4 mM oleate bound to 0.55 mM BSA, or 0.4 mM palmitate and 0.4 mM oleate bound to 0.55 mM BSA. For assessment of the acute effect of fatty acids on BMMSC energy metabolism, BMMSCs were only given these treatments while energy metabolism was being measured. Up until these assays began BMMSCs were only exposed to standard cell culture media. Palmitate or oleate alone did not affect glycolysis, glucose oxidation, or oleate oxidation rates (Fig. 4). However, combined treatment with palmitate and oleate did significantly reduce glucose oxidation rates (Fig. 4B), a condition known to be associated with increased cell proliferation [9]. As expected, palmitate and oleate inhibited each other’s uptake (Fig. 4E,F), although neither inhibited each other’s oxidation (Fig. 4C,E). This suggests that oleate resulted in a better coupling of palmitate uptake to palmitate oxidation, resulting in less palmitate entering other cellular pathways.

### Chronic effects of the fatty acids palmitate and oleate on BMMSC energy metabolism

**Fig 5:**
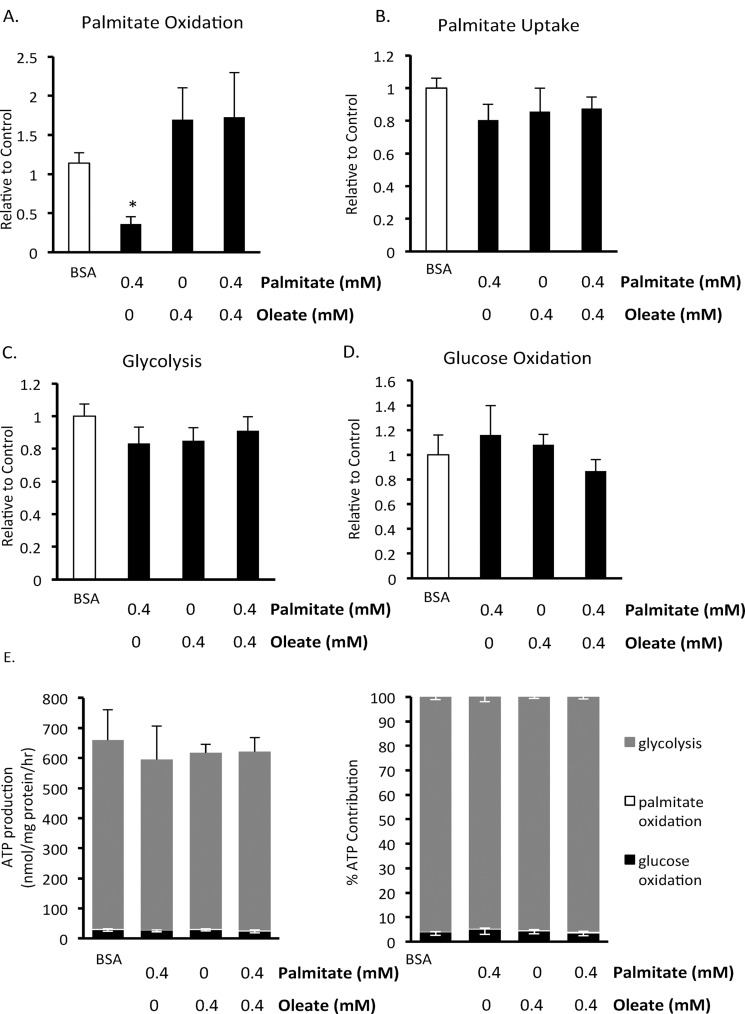
Effect of 24 hour exposure to fatty acids on human BMMSC energy metabolism. A) Palmitate oxidation, B) palmitate uptake, C) glycolysis, and D) glucose oxidation were measured in human BMMSCs that had been treated for 24 hr with either 0.55 mM albumin (BSA group) or 0.55 mM albumin and 0.4 mM palmitate and/or 0.4 mM oleate prior to these metabolism measurements being made. n = 5–8 The graphs indicate which groups were exposed to these different treatments for the 24 hr prior to the metabolism measurements. During each assay all groups were given Krebs buffer supplemented with 5 mM glucose and 0.4 mM palmitate bound to 0.55 mM albumin. In addition, the Krebs buffer was supplemented with either [U-^14^C]glucose, [1–^14^C]palmitate, or [5–^3^H]glucose for the measurement of glucose oxidation, palmitate oxidation and uptake, or glycolysis, respectively. E) The contribution of metabolic pathways to ATP production were calculated from the metabolic rate results. * Significantly different from all groups. Values are shown as mean ± SEM.

Since chronic exposure of BMMSCs results in decreased cell viability, we also investigated what effect exposure of human BMMSCs to fatty acids had on energy metabolism (Fig. 5). Since 72 hr exposure of BMMSCs to palmitate resulted in a substantial decrease in cell viability (Fig. 1), cells were treated with palmitate for 24 hr prior to measurements of energy metabolism, a time period where no decrease in cell viability was observed. BMMSCs were treated for 24 hr with 5 mM glucose and either 0.55 mM BSA, 0.4 mM palmitate bound to 0.55 mM BSA, 0.4 mM oleate bound to 0.55 mM BSA, or 0.4 mM palmitate and 0.4 mM oleate bound to 0.55 mM BSA. Interestingly, chronic palmitate treatment decreases palmitate oxidation rates (Fig. 5A), without altering palmitate uptake rates (Fig. 5B). Chronic exposure to oleate prevents the decrease in palmitate oxidation without altering palmitate uptake rates (Fig. 5A,B), thereby improving the coupling between palmitate uptake and oxidation. Neither chronic palmitate and/or oleate treatment affects BMMSC glucose oxidation or glycolysis (Fig. 5C,D). Overall, glycolysis remains the major source of ATP production in BMMSCs which are chronically exposed to palmitate and/or oleate (Fig. 5E,F).

### Chronic effects of palmitate and/or oleate on BMMSC expression of proteins involved in glycolysis and oxidative metabolism

**Fig 6:**
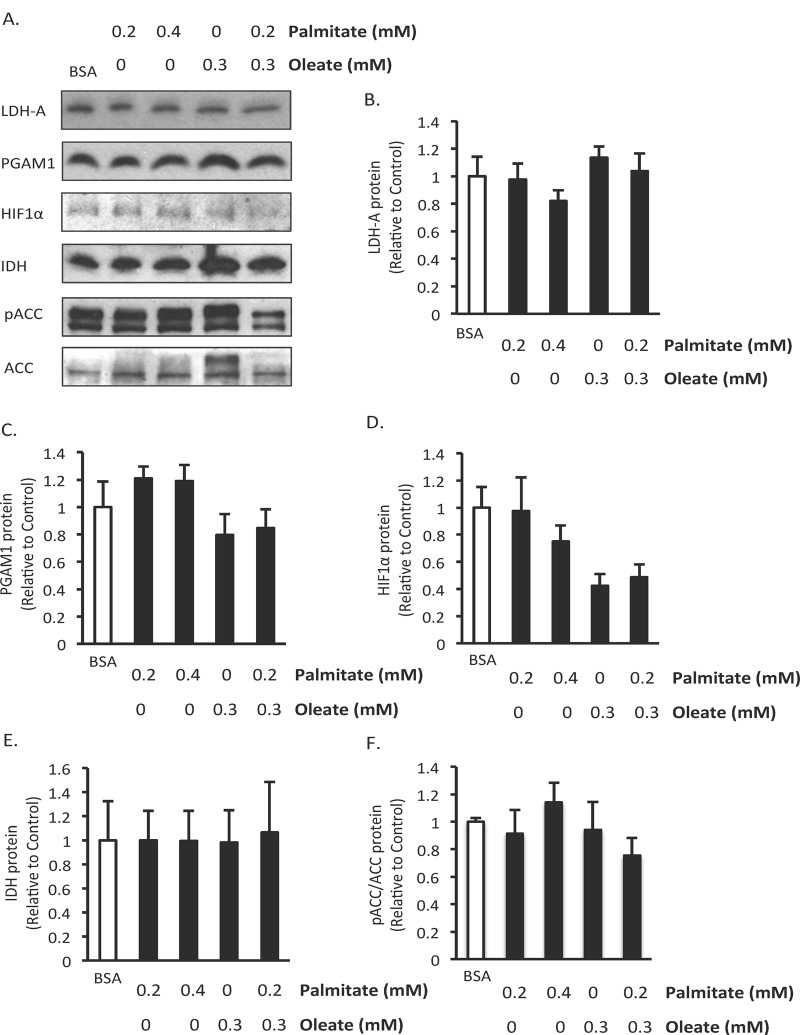
Effect of 24 hr exposure to fatty acids on expression of proteins involved in energy metabolism. A) Representative western blots, B) Lactate dehydrogenase A (LDHA), C) Phosphoglycerate mutase 1 (PGAM1), D) Hypoxia inducible factor 1α (HIF1α), E) Isocitrate dehydrogenase (IDH), F) phospho Acetyl CoA carboxylase (ACC)/ACC protein expression. n = 5–6 Protein expression was measured in human bone marrow mesenchymal stem cells (BMMSCs) treated for 24 hr with indicated treatments. The BSA group was treated with media supplemented with 0.55 mM albumin. All fatty acid treated groups were also treated with media supplemented with 0.55 mM albumin in addition to the type and amount of fatty acid indicated in the figures. Values are shown as mean ± SEM.

To further investigate the effect of fatty acids on BMMSC energy metabolism we assessed the effect of 24 hr treatment with palmitate and/or oleate on the expression of proteins involved in glycolysis and oxidative metabolism. We chose this length of treatment because it was long enough to potentially observe changes in protein expression but soon enough that there would still be cells present to make measurements in. No significant changes occurred in the protein expression of PGAM1 and LDH-A, two proteins involved in glycolysis (Fig. 6A,B). Interestingly, HIF1α protein expression, a key transcription factor involved in regulating glycolysis, trends towards being reduced in both groups treated with oleate but palmitate alone did not affect its expression (Fig. 6C). Isocitrate dehydrogenase, an enzyme involve in the TCA cycle, was not affected by any of the treatments (Fig. 6D). There were also no changes in ACC expression or phosphorylation of ACC (Fig. 6E), a key enzyme involved in the synthesis of malonyl CoA, which is a potent inhibitor of mitochondrial fatty acid uptake and oxidation. This suggests that regulation of ACC is not an explanation for the reduction in palmitate oxidation observed following 24 hr treatment with palmitate.

## Discussion

This is the first study to directly determine the energy metabolic rate profile of human BMMSCs. We confirmed the previous assumption that BMMSCs derive most of their ATP from glycolysis (>97%) (Table 1). This finding is in agreement with indirect measurements of energy metabolism including those showing elevated lactate levels and low oxygen consumption rates in several types of stem cells including mesenchymal, embryonic, and induced pluripotent stem cells [8,32–34]. In support of high rates of glycolysis being important for pluripotency, studies have shown that osteogenic differentiation of mesenchymal stem cells and ESC-to-cardiomyocyte differentiation are accompanied by a decline in lactate production [8,33]. We also examined the effect of various fatty acids on the energy substrate metabolism, survival, and proliferation of human BMMSCs. We show that physiologically relevant levels of saturated fatty acids induce BMMSC death and decrease BMMSC proliferation, effects which are prevented by the unsaturated fatty acid oleate. These experiments were designed to assess the effect of levels of fatty acids present in the circulation on BMMSCs. It will be interesting in the future to also assess the effect of the level of fatty acids present in the bone marrow on BMMSC survival. We also show that decreasing saturated fatty acid oxidation may induce BMMSC death. This has important implications on the therapeutic strategy of using BMMSCs for tissue regeneration, and suggests that strategies should be implemented that minimize circulating saturated fatty acid levels during the therapy.

Fatty acids have previously been reported to affect cell survival. Saturated fatty acids have specifically been reported to induce death in many cell types, including BMMSCs [35–37]. However, many of these studies used a level of albumin that is much lower than that present in the circulation (0.55 mM). The use of this low level of albumin results in cells used in such studies being exposed to an artificially high level of palmitate [28]. Therefore, in our experiments the level of albumin we always used was 0.55 mM. We found that physiologically relevant levels of palmitate ranging from levels present under fed to fasting conditions induce human BMMSC death while oleate, an unsaturated fatty acid, does not (Figs. 1 and 2). These results disagree with a previous study by Smith et al [38] that reported that oleate induces BMMSC death. In fact, we show that oleate can actually protect BMMSCs from palmitate-induced cell death. It is possible that the discrepancy in Smith et al’s findings and ours are simply due to Smith et al exposing BMMSCs to relatively higher levels of oleate (due to the fact that the albumin concentration used was low) [38]. Regardless, the data highlight the need to carefully consider both the fatty acid concentration and albumin concentration to which the BMMSC is exposed during any attempts at stem cell therapy.

Fatty acids can regulate flux through energy metabolic pathways, and may thereby regulate cell survival. The survival and proliferation of cells with high glycolytic rates tends to be positively correlated with glycolysis [9,15]. In other cell types a process referred to as the glucose-fatty acid cycle, or the Randle cycle, has been observed, where increased fatty acid oxidation can inhibit glucose oxidation and glycolysis [39]. However, in the heart it has frequently been reported that elevating fatty acid oxidation results in uncoupling of glycolysis from glucose oxidation, due to a greater inhibition of glucose oxidation than glycolysis [40]. In agreement with this, inhibiting fatty acid oxidation via MCD inhibition results in pulmonary artery smooth muscle cell apoptosis and decreased proliferation [10]. This is probably detrimental to these cells because decreasing palmitate oxidation likely results in an improved coupling of glycolysis to glucose oxidation. Therefore, this link between fatty acid oxidation and glucose metabolism could explain why fatty acid oxidation seems to regulate cell proliferation and survival.

An alternative explanation for the effects of fatty acids on cell survival is that fatty acid oxidation could be beneficial independent of its effects on glycolysis. It has been suggested that under conditions where glycolysis is reduced fatty acid oxidation can be used by cancer cells for energy production [25]. We therefore decided to determine whether fatty acids inhibit human BMMSC glucose metabolism and induce BMMSC death via modulation of glucose and fatty acid energy metabolism. Acute exposure to palmitate and/or oleate did not affect glycolysis or fatty acid oxidation rates (Fig. 4). However, combined acute exposure to palmitate and oleate did reduce glucose oxidation (Fig. 4B). These results indicate that the Randle Cycle exists at least to some extent in human BMMSCs. Following chronic treatment with palmitate and/or oleate we observed that only palmitate exposure reduced palmitate oxidation rates (Fig. 5). Interestingly, combined treatment with oleate, which prevented palmitate-induced death, prevented this reduction in palmitate oxidation (Fig. 5). Neither palmitate or oleate affected the expression of proteins involved in oxidative metabolism or glycolysis that we measured (Fig. 6). These palmitate oxidation results agree with a previous report showing that 20 hr exposure of neonatal cardiac myocytes to palmitate induced apoptosis and decreased palmitate oxidation rates [41]. This suggests that palmitate induces BMMSC death via inhibition of palmitate oxidation and that oleate is protective because it prevents palmitate oxidation from decreasing. This is supported by a previous study in BMMSCs in which AICAR (an activator of AMPK and fatty acid oxidation) prevented palmitate-induced death [35]. However, we did not observe a change in phosphorylation of ACC, an indicator of AMPK activity and an important pathway by which AMPK increases fatty acid oxidation. This may not actually be that surprising, since AMPK activation can decrease proliferation but as we show here oleate protects against the drop in proliferation induced by palmitate [42]. It is still a possibility, however, that a reduction in glycolysis may be involved in palmitate-induced BMMSC death, but changes in glycolysis that occur in response to 24 hr treatment with palmitate and/or oleate were masked by switching all groups to the same buffer during the measurement of glycolysis rates.

Oleate had a dramatic effect of preventing palmitate-induced BMMSC death. This may have occurred secondary to inhibiting palmitate uptake. Acutely, oleate and palmitate reduced each other’s uptake (Fig. 4). However, after 24 hr of exposure to palmitate and/or oleate, palmitate uptake was not different between groups (Fig. 5). It is still possible, however, that oleate did in fact reduce palmitate uptake at 24 hr but it was an acute effect and therefore was not measured (since during the assay the cells in all groups were exposed to 0.4 mM palmitate). Therefore, oleate may be at least partially protecting against palmitate-induced cell death by reducing intracellular palmitate levels by decreasing palmitate uptake.

Another potential mechanism for palmitate-induced cell death in the BMMSCs is the potential involvement of ceramides. Elevated levels of ceramides are able to induce death in a number of different cell types [43]. The fact that saturated fatty acids (palmitate and stearate), which are ceramide substrates, induced BMMSC death while oleate, an unsaturated fatty acid which is not a ceramide substrate, does not induce BMMSC death suggests that ceramides could be involved in saturated fatty acid-induced BMMSC death. In addition, chronic exposure to palmitate reduces fatty acid oxidation, which could result in a redirection of palmitate into ceramides. Further, oleate prevented this drop in fatty acid oxidation and decreased palmitate uptake, which could decrease ceramide production by reducing the amount of palmitate present to be used in ceramide production. In fact, elevated ceramide levels accompany the palmitate-induced reduction in fatty acid oxidation in neonatal cardiac myocytes [41]. Unfortunately, experimental conditions precluded us from measuring ceramide levels in these cells. However, there is evidence that palmitate at least does not always work through ceramides to induce cell death [36,37,43].

## Conclusion

We demonstrate that human BMMSC energy production is predominantly derived from glycolysis, and we show that modulation of energy metabolism is important in the proliferation and survival of human BMMSCs. In particular, physiologically relevant levels of saturated fatty acids reduce BMMSC proliferation and induce BMMSC apoptosis, all effects that can be prevented by oleate. The decrease in saturated fatty acid oxidation induced by chronic exposure to palmitate may be involved in these deleterious effects of palmitate on BMMSCs. These observations indicate that saturated fatty acids could be contributing to the low *in vivo* survival of BMMSCs, and therefore to the disappointing results of stem cell therapy clinical trials including those focused on treating heart and pulmonary diseases [1–6,44–46].
